# Deferred Action for Childhood Arrivals (DACA) medical students – an examination of their journey and experiences as medical students in limbo

**DOI:** 10.1186/s12909-021-02787-5

**Published:** 2021-06-28

**Authors:** Christina Gillezeau, Wil Lieberman-Cribbin, Kristin Bevilacqua, Julio Ramos, Naomi Alpert, Raja Flores, Rebecca M. Schwartz, Emanuela Taioli

**Affiliations:** 1 Institute for Translational Epidemiology and Department of Population Health Science and Policy, Icahn School of Medicine at Mount Sinai, 1 Gustave L. Levy Place, Box 1133, New York, NY 10029 USA; 2 Department of Population, Family and Reproductive Health, Johns Hopkins Bloomberg School of Public Health, 615 N Wolfe St, Baltimore, MD 21205 USA; 3 Department of Thoracic Surgery, Icahn School of Medicine at Mount Sinai, New York, NY 10029 USA; 4 Department of Occupational Medicine, Epidemiology and Prevention, Zucker School of Medicine at Hofstra/Northwell, Great Neck, NY 11021 USA

**Keywords:** DACA, Medical education, Survey data, Qualitative interviews

## Abstract

**Background:**

Although the value of DACA medical students has been hypothesized, no data are available on their contribution to US healthcare. While the exact number of DACA recipients in medical school is unknown, DACA medical students are projected to represent an increasing proportion of physicians in the future. The current literature on DACA students has not analyzed the experiences of these students.

**Methods:**

A mixed-methods study on the career intentions and experiences of DACA medical students was performed utilizing survey data and in-depth interviews. The academic performance of a convenience sample of DACA medical students was compared to that of matriculated medical students from corresponding medical schools, national averages, and first-year residents according to specialty.

**Results:**

Thirty-three DACA medical students completed the survey and five participated in a qualitative interview. The average undergraduate GPA (SD) of the DACA medical student sample was 3.7 (0.3), the same as the national GPA of 2017–2018 matriculated medical students. The most common intended residency programs were Internal Medicine (27.2%), Emergency Medicine (15.2%), and Family Medicine (9.1%). In interviews, DACA students discussed their motivation for pursuing medicine, barriers and facilitators that they faced in attending medical school, their experiences as medical students, and their future plans.

**Conclusions:**

The intent of this sample to pursue medical specialties in which there is a growing need further exemplifies the unique value of these students. It is vital to protect the status of DACA recipients and realize the contributions that DACA physicians provide to US healthcare.

**Supplementary Information:**

The online version contains supplementary material available at 10.1186/s12909-021-02787-5.

## Background

In June 2012, President Barack Obama signed an executive order enacting protections for undocumented immigrants brought to the United States as minors. The policy, known as the Deferred Action for Childhood Arrivals (DACA), offers a path forward for eligible undocumented young people brought to the United States as children to remain in the country, receive work authorization, and participate in the Social Security Program. To date, there are 649,070 active DACA recipients [1]. In 2017, the Trump Administration sought to end DACA through executive order but was blocked by two federal appellate courts, allowing previous recipients to renew their status [2]. In June 2020, the Supreme Court of the United States ruled on this matter and rejected the effort to deconstruct and abolish the DACA program. However, as this ruling does not prevent against future policy regarding DACA, the status of these DACA recipients living in the United States remains in limbo. President Joe Biden has promised to reinstate DACA and ensure that DACA recipients are eligible for federal student aid, but has not yet implemented a plan for citizenship for DACA recipients [3]. Without concrete legislation, DACA recipients may remain vulnerable to policy changes in future administrations.

Educational opportunities are an integral part of the DACA program. DACA status allows recipients to apply for a Student Aid Report, which is used to determine the amount of financial aid that students might be able to receive based on their family’s income, and, in some states, to be eligible for in-state tuition rates and educational grants [4]. As of September 2017, 62% of DACA recipients who were not active in the labor force were enrolled in school [5]. While 18% of DACA recipients were in post-secondary education, the precise number of DACA participants in medical school is unknown, with estimates varying from 70 to 100 recipients currently enrolled [6, 7]. Although no formal policy has been implemented by US medical schools for addressing applications from prospective DACA students [8], from 2012 to 2014, the Association of American Medical Colleges (AAMC) reported an 8-fold increase in the number of medical school applicants indicating DACA status [8]. Based on these estimates, DACA medical students are projected to account for 5400 to 31,860 new, underrepresented minority physicians entering the workforce over the next few decades [9]. We have previously conveyed the unique contributions of a distinct socioeconomic, cultural and linguistically diverse sample of DACA medical students [10]. The research literature on DACA students however has largely focused on their potential contributions to society without analyzing the experiences of the students themselves. Here, we utilize survey methodology to understand academic performance and career intentions of DACA medical students and we utilize individual in-depth interviews on a subgroup of the survey participants to explore their life experiences in order to elucidate the ways in which these experiences informed their motivations for pursuing medicine and their future goals.

## Methods

### Survey

An anonymous survey was conducted between April 2018 and July 2018; details have been previously described [10]. In brief, students were recruited through word-of-mouth from other DACA medical students and by advertising the survey through a grassroots organization that provides resources to DACA recipients who want to pursue a career in the health field. Participants provided information about their education, including undergraduate school attended, Grade Point Averages (GPA), major(s), medical school attended, Medical College Admissions Test (MCAT) scores, and way(s) of financing medical school. Participants also recorded what medical field they intend to pursue. DACA medical students also indicated if they would be interested in participating in semi-structured interviews. Data was completely de-identified thereby prohibiting the ability to identify any individual participant as a DACA recipient.

### Statistical analysis

Self-reported MCAT scores from DACA medical students were converted into percentile ranks according to the distribution of MCAT scores from the appropriate test month and year, utilizing data from the AAMC [11]. To compare DACA medical student MCAT scores and GPAs to those of matriculated medical students, MCAT scores and GPAs were obtained from school admission websites. When multiple participants reported attending the same school, an average self-reported MCAT percentile and average self-reported GPA was calculated. For further comparison, national MCAT and GPA data of 2017–2018 matriculated students was obtained from the AAMC [11].

### Interviews

Of the 33 students who completed the survey, six students indicated interest in completing a qualitative interview and were contacted in July 2019. Five students, two of whom were female and three of whom were male, were recruited via convenience sampling based on providing agreement to be contacted. One additional person was initially interested in participating but ultimately did not respond after three attempts were made to set up an interview time. Given the low response rate, saturation was not reached and the qualitative data is considered preliminary. Interviews were conducted via phone by two masters-level research assistants, one male and one female, trained in qualitative interviewing. Interviews were recorded with the consent of the participant. Each interview began with the administration of informed consent. The lead qualitative investigator then followed a semi-structured interview guide (Supplementary Appendix) to better understand barriers DACA students faced in applying for medical school, as well as their experiences in medical school and their future goals. All interviews were audiotaped and transcribed verbatim with any identifying details including names, cities, or schools removed to protect the participants’ identities. Participants were reimbursed with a $20 gift card for their time.

Utilizing an inductive approach, the transcribed data were analyzed by four study team members who reviewed transcripts for emergent themes. Codes were then created to characterize those emergent themes. The codes were revised in an iterative process to ensure coverage and reliability between coders. Each interview was coded by two study team members and coding inconsistencies were reconciled by the lead qualitative investigator. Analyses were conducted using NVivo Qualitative Data Analysis software (NVivo. Version 12. QSR International; 2018).

## Results

Thirty-three DACA medical students completed the survey and their data were included in the quantitative analysis. Twenty of these participants were male (61%), and participants were on average (SD) 25.7 years (2.5 years) old at the time of survey completion. The average age (SD) at arrival in the US was 5.9 years (3.3 years). The study sample was Mixed Race (42.4%), Asian/Pacific Islander (36.4%), White alone (18.2%), and African American alone (3.0%). Sixty-one percent of participants indicated Latino/Hispanic ethnicity, and the sample was mostly born in North America (30.3%), Asia (30.3%), and South America (24.2%) rather than Central America (9.1%) or Africa (6.1%).

**Fig. 1:**
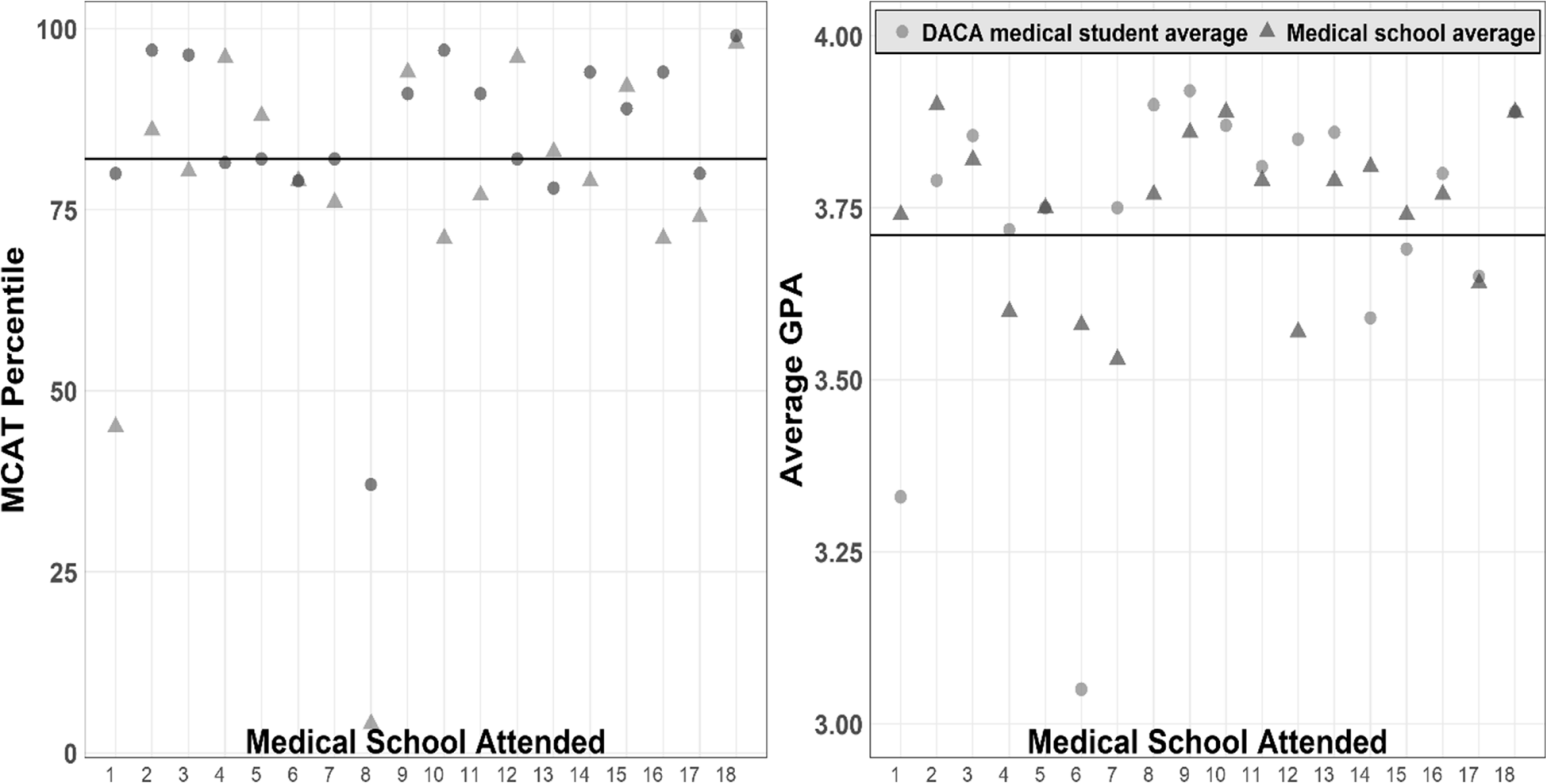
MCAT percentiles (left) and GPAs (right) of the DACA sample compared to the corresponding medical school attended and national averages. MCAT and GPA data was obtained from medical school admissions profiles. MCAT and GPA data from DACA respondents from the same medical school were averaged. The horizontal line represents the national average MCAT percentile and GPA of matriculated medical students in 2017–2018 [11]. Medical schools attended were kept confidential due to the sensitive nature of that information

The average undergraduate GPA (SD) of the sample was 3.7 (0.3) on a 4.0 scale, while the average MCAT score was 33.1 (3.2) out of 45 for the pre-2015 MCAT scoring and 507.7 (9.4) out of 528 for the post-2015 MCAT scoring. The average MCAT percentile for all years was 80.1 (20.1) (Fig. 1). The median MCAT percentile was 84.5. Among medical schools that the DACA sample attended, the average MCAT percentile was 85.2 (12.7), the median MCAT percentile was 83.0, and the average GPA was 3.7 (0.1). Nationally, the average GPA of 2017–2018 matriculated students was 3.7 (0.3), while the average MCAT score was 510.4 (6.6), corresponding to the 82nd percentile.

At the time of survey completion, more students were in their first (46.9%) and second (28.1%) years of medical school rather than their third (21.9%) or fourth (3.1%). The most common intended residency programs were Internal Medicine (27.2%), Emergency Medicine (15.2%), and Family Medicine (9.1%) (Table 1). Compared to national MCAT data of first-year residents [13], MCAT percentiles of the DACA sample were consistently above the 75th percentile for all intended residency programs: Pathology (96.0), Anesthesiology (94.5), Psychiatry (94.0), Pediatrics (91.0), Internal Medicine/Pediatrics (88.0), Emergency Medicine (86.3), Family Medicine (83.0), Neurological Surgery (77.0), and Internal Medicine (76.0).

**Table 1:** Intended residency training program of the DACA sample

Residency	Number of Responses (Percent)	Percentage Change in the number of first-year residents and fellows by specialty (2010–2015)^**a**^
Anesthesiology	2 (6.1)	−14.4
Emergency Medicine	5 (15.2)	7.8
Family Medicine	3 (9.1)	0.9
General Surgery	2 (6.1)	5.1
Internal Medicine	9 (27.3)	2.5
Neurological Surgery	1 (3.0)	1.0
Obstetrician-gynecologist	1 (3.0)	−3.6
Plastic Surgery	1 (3.0)	21.7
Pathology	1 (3.0)	−7.0
Pediatrics	2 (6.1)	0.2
Psychiatry	1 (3.0)	5.3
Undecided	4 (12.1)	–
Urology	1 (3.0)	0.7
Total	33 (100.0)	20.2

^a^ Percentage Change in the number of first-year residents and fellows by specialty was sourced from GME Track [12]

### Motivations for pursuing medicine

Four of the five participants described a personal calling to medicine from a young age. Students framed their motivations for pursuing a career in medicine in terms of their own experiences growing up without documentation. They cited lack of insurance, barriers to accessing care, and challenges communicating with healthcare providers as common experiences after migrating to the United States. One student recalled,

[…] *being undocumented, definitely like growing up without health insurance, and this seeing the --in the community that I grew up with basically has different, different access to care. Like, even while young, I remember volunteering at these […] at my mom's organization the most, and the, the community members-- like, you know, we'll be finding out that they had, like, these chronic conditions right then and there, and not had-- and it wasn't prevented by their PCP [primary care provider] because they didn't have access to, like, insurance, so they didn't have PCP -Participant 1, Medical School Year 1*

Another student described serving as an interpreter for her mother while undergoing treatment for breast cancer as a formative experience.

*So she, the reason, so she had breast cancer in 2005. And it, the reason that I, I think, I had the idea of getting into medicine is because I was the only one in my house that spoke English, that would accompany her to doctor's appointments. And I saw how the oncologist affected my mother, and then how my mother then affected our family. So I realized that doctors have a special role in taking care of families in the community. And I think I wa-- I'm pursuing that, sort of like that relationship with the, with whatever community it, that I end up in. -Participant 4, Medical School Year 2*

Four of the participants also considered their experiences when deciding on a specialty within medicine and also the setting in which they hoped to practice medicine after residency. All students described hoping to work with undocumented, uninsured or underinsured populations, mainly in a primary care capacity. Those students who hoped to pursue a more specialized field of medicine still cited a desire to work with underserved communities.

*[P]eople who are undocumented, people who are poor, like, will still need neurologists* […] *Like, although, like, it's a very niche, like, group* […] *if I ended up doing, like, neuro-oncology, but I think that, like, I would want to help patients that are undocumented or uninsured, that, like, get brain cancer. 'Cause, you know, it's not, like, not like brain cancer, like, just remains in a certain population, like it targets all, so then by that, like, uh, I feel like I'm still kind of holding true to, like, why I wanted to pursue medicine to help those people -Participant 1, Medical School Year1*

Overall, students emphasized their intentions to enter into fields where they feel that they can do the most good for their community or society at large. Participants placed their immigration status central to their goals to serve the undocumented communities.

### Barriers and facilitators to pursing medicine

Though the majority of students recalled hoping to pursue medicine from a young age, they described their status as undocumented as an initial barrier to attending medical school. One student recalled:

*[Y]ou know, [I] was definitely told that I couldn't go to medical school when I was younger, so that was, like, a dream that I kinda had to give up-give up, uh, when I was younger because I was told that it just wasn't gonna be possible unless I, like, married for my papers or whatever. This was obviously all before DACA, so I guess it was, like, a little bit different then. -Participant 1*

Similarly, students recalled realizing their dream to pursue medicine would be possible though DACA.

*Um, I can't imagine still being undocumented-- I don't even know if I could-- I would be in medical school. Um, because they-- yeah. I just-- at the-- at the time-- I think at the time I was applying, [medical school] specifically-it was only taking DACA, not undocumented. I think their policies are starting to change, um, but they-- I don't even think I would be here. -Participant 5, Medical School Year 3*

When asked what their life would look like without DACA one student responded:

[…] *for one, like, would not have, like, been able to fill out my application as well as I did. 'Cause, like, once I got DACA, like, I was able to, you know, work. I did research at [university], I was able to work at [university]. All these things that was like, if I had not done that, like, I would not have been able to [apply to medical school]. Um, 'cause, like, you know, work permits, part of DACA is what, like, helped me the most. -Participant 1 Medical School Year 1*

Despite the opportunities afforded by their DACA status, students still faced formidable barriers in applying to and attending medical school. The primary barriers cited by students were financial. For example, many medical schools consider undocumented students international, making them ineligible for student loans and other forms of financial aid. Even with DACA status, state residency rules impacted access to financial aid. After being admitted to a prestigious medical school one student recalled:

*And then my other fear was that even if I got into a school, um, that I really wanted to go to that I wouldn't be able to go because of matriculation, um, requirements, uh, for citizenship or residency status, or because of financial aid. Um, and actually did happen. So I got into [university]* […] *But they essentially said that* […] *they couldn't get me any private loans whatsoever. And so it turned out that I, I couldn't go there even though I had gotten in. Um, a-and then mostly it had to do with the fact that I'm documented and out of state. Because if you're in state, they have, um, a rule* […] *that if you went to high school in the state, you can get, you know, tuition assistance. -Participant 2, Medical School Year 2*

Similarly, differing policies around admission of DACA students created confusion for students when completing applications. Students described contacting schools before applying for clarification on policies, but often being left with lingering confusion. One student described applying to medical school three times as an in-state resident in the state where they were raised, before ultimately attending school out-of-state.

[…] *so I went through the whole interview process like a regular resident student, and then once I was admitted, I couldn't provide the supporting documents, so they happened to, they happened to, uh, like, d-- uh, retract their decision. Ah. And this actually happened three times, um, just because I, I didn't really know what was going on. And it was only until, like, the third time that I realized I needed to call and see that this, this doesn't happen again. Make sure that they knew. And that, yeah, and then I was very upfront in all my interviews, saying that I, I was an, uh, undocumented student. Um, and even then, I still managed to become accepted, but then there was a discrepancy between the interviewers and the, I guess, the administration of the school* […] *I was still accepted, and then they denied me again after that I couldn't provide* […] *permanent status. -Participant 4, Medical School Year 2*

Despite this experience, the student managed to find a mentor who helped them better navigate the application process.

*Um, and then it was only until I did some research online that I found another mentor that kinda like guided me and told me that I needed to apply to schools out of my state, um, and those schools would be DACA-friendly and would accept me and would actually provide even some financial aid. -Participant 4, Medical School Year 2*

Support and guidance from mentors was a common theme in our interviews before students applied to medical school. However, when applying to medical school, mentorship was described as an instrumental part of the process. Mentors included both individual school counselors as well as organizations created by and for DACA recipients pursing medicine. One such organization was described by four of five interviewees and provided resources including a living document with information about DACA-friendly medical schools.

*And I found this [Facebook group], and they kind of had a lot of tips. Uh, I think [medical school] also had an event, um, for, um, [the Facebook group], and I remember going to it and-- so then, I found the website and these were the schools that were friendly and I'm like, ‘These are the schools that I'm gonna apply to’. -Participant 3,Medical School Year 2*

In addition to instrumental support, students described social support from family, mentors, organizations and other DACA students as an integral part of their academic journey. When describing apply to college one interviewee recalled:

*Um, luckily, I had a mentor who didn't necessarily know how to he-help me, but they were there emotionally, which I think was also very critical for my, for my admission into college. She told me, "I don't know how, but we're gonna get you there." And I think that sort of sentiment has followed me even to this day. After I'd completed college, I tried to do the same thing for, uh, medical school. They don't how, um, but I knew that if I tried hard enough, I would do it. -Participant 4, Medical School Year 2*

### Medical school experience

In addition to the normal stressors associated with medical school, DACA recipients described unique stressors related to recent policy changes surrounding the DACA. Students described their experiences learning about President Trump’s 2017 order to end the DACA program, including during important milestones during their medical education. One student found out about the order just before their white coat ceremony, an important rite of passage for medical students.

*I remember that pretty vividly because my first semester was really, um, scary. […] I remember the day that they, you know, made the announcement, I-- it was actually the day […] like people essentially does the ritual to walk through the medical gates. And I remember that, like, the news have come up, that everyone was already lined up. All the grad students and, you know, just kinda having to go through that realizing that, you know, I was walking into, like, my medical school career knowing that, um, I wouldn't be able to practice, you know. […]And so it's kinda like a pretty vivid picture where, you know, I was walking into the gates, you know, being allowed into medical school. But then, knowing that if I-- you know, I was going to come out of those gates four years later and not be able to practice medicine. -Participant 2, Medical School Year 2*

Another student recalls the impact of the announcement on their grades.

*So it was my first* […] *semester of medical school. It was-- it was definitely stressful.* […] *I think, distracted me a bit from studies. Um, I remember my first med school exam. Uh, I actually thought I failed it. Um, but there was a curve, and it turned out I was fine. But I was still on-- scoring on the lower end. Um, but, uh, at that time, yeah. It was-- I was just having trouble sort of concentrating on, on studying, just because I, I guess I didn't know what was to lie ahead. -Participant 5, Medical School Year 3*

All students spoke about what the order meant for the future ability to apply to residency programs and ultimately, to practice medicine in the United States.

*Um, I was actually in my anatomy class, in, doing a dissection in, in a ca-- uh, on a cadaver […]we couldn't access our phones, but I, immediately afterward I scrubbed out […] and went online to check what the decision was. And immediately, I mean, it, I don't even know, because I, we were anticipating the decision and it was just a matter of, like, him announcing, announcing it. Um, so I th-think I was mentally prepared. And I was, I was, essentially just devastated because I had, I, I really didn't know what that, eh, that decision meant. Um, but at face value, it meant that all the work that I had to get to this point now could immediately, by one signature, be taken away. -Participant 4 Medical School Year 2*

Students also spoke about decision-making related to whether they should disclose their status and to whom. Many of the students described disclosing their status as an important form of activism in the face of ongoing challenges to the DACA program and a way to cope with the uncertainty they face as DACA recipients. Students acknowledged that as medical students, they are in a unique place of privilege from where they can share their stories and highlight the potential benefits of DACA to society more broadly.

[…] *it was mostly because I realized that as a medical student* […] *we kinda feel that, you know, it's unfortunate that it is this way. But I think that, you know, like newspapers and just like American society see some* […] *type of value in the fact that we are in medical school. And we felt that maybe that was something we could use to the advantage of, you know, like the DACA program and just other people who, whose stories may be would not had been valued. Even though they're equally as hardworking, if not more. And so I think that that was kind of a moment where we all came together and, like, and a lot of people stepped up to tell their stories. -Participant 2, Medical School Year 2*

Though they acknowledged their privilege as medical students, interviewees also cautioned against this view of who might be worthy of DACA status or of citizenship.

[…] *if they wanna see it in, in a more economical way, you know, they can look at the numbers of, um, you know, tax, uh, paying or whatever it is. But I think I would also caution them to keep in my mind that, uh, that people are people and they shouldn't be valued by-- or they shouldn't be measured by their productivity or who they are or whatever titles they have next to the name. But that, you know, at the end of the day, we're humans and we deserve respect, um, as much as, you know, anyone else. -Participant 2, Medical School Year 2*

### Future plans

With DACA’s fate still uncertain, many students described fears around their ability to fulfill lifelong dreams of practicing medicine. Students described beginning to make plans surrounding how to continue their training should DACA end or should they face deportation.

*Definitely run through different scenarios. Um, I mean, I, I would find a way to finish my degree, which, hopefully the school would be able to help me with. And then, I've seen a lot of people do their, their, um, residencies abroad and then come back. Or, um, they do a lot of, like, consulting work. That's kind of like what the worst scenario would look like. -Participant 3, Medical School Year 3*

Another student concluded that the end of DACA would not be the end of pursuing their goals, but rather an extension of the same challenges they have experienced as an undocumented person in the United States.

*[…] specifically for undocumented students that are in school. Um, everything could end just by one vote […] but I think in sort of like building a generation of undocumented students that have been, that have grown with DACA and have a little bit more access to, uh, these opportunities has empowered them […] it has empowered me to continue pursuing whatever goal I have. I sorta was told that we, that it was not possible, and fortunately, I am now in a position in which I can say that I have accomplished one of my goals, being in medical school. So that idea of if you work and you don't give up, it can come true in the future, become true. Um, sorta like, even though we may not have DACA status, the fight towards accomplishing our goals would not end. So in the sense, in that sense, I think that the idea of the American Dream is not necessarily completely false, because the, the values are still there. -Participant 4, Medical School Year 2*

## Discussion

This is the first work to discuss the experiences of DACA medical students while examining their scholastic achievement and career objectives. Results indicated that DACA medical students have similar GPAs and MCAT scores when compared with other matriculated medical students, both nationwide and within their given medical school, and they appear to have greater motivations to enter primary care specialties. The intent of this sample to pursue medical specialties in which there is a growing need further exemplifies the ways in which diverse experiences among medical professionals can benefit the field more broadly.

When asked to describe their motivations for becoming physicians, all of the interviewees spoke of a desire to give back to their community, whether that was the undocumented immigrant community or underserved communities more broadly. Many of them described a desire to pursue internal medicine because they felt it would provide them with an avenue to work with undocumented patients, but even those who planned to pursue more specific specialties considered how to ensure that their work touched the lives of undocumented patients as well.

DACA medical students identified legal, institutional, and financial issues as the primary barriers they had to surmount in order to attend medical school. The largest single barrier faced by all DACA medical students prior to the implementation of the DACA program was a lack of documentation, without which, medical students could not apply to residency and thus were not able to become practicing physicians. This meant that, given the financial investment made in training new physicians by medical schools, medical schools were unwilling to admit undocumented students knowing that this investment could not be recouped [13]. DACA changed that calculation, and offered a path to becoming a licensed physician for recipients.

Even after the legal barriers were removed when the DACA program was enacted, medical schools still remained inconsistent and unclear regarding their admissions policies on accepting DACA students. Among our sample, students described being accepted to several different institutions before having their acceptance rescinded due to their DACA status, despite having been open about their status from the beginning. One argument brought against DACA is that recipients take positions that would otherwise be filled by American citizens. However, the current system employed by medical schools indicates that this is not the case [14]. Instead, institutions with clear DACA student acceptance policies often limited the number of DACA students that they can accept to one or two students per year, creating a great deal of competition for those limited slots.

Finally, finances were a major barrier to attending medical school for DACA students because they were ineligible for federal student loans. The median debt for all medical students in the class of 2020 was $200,000, which given that the median 4-year cost of attendance for medical students was $259,347 for public schools and $346,955 for private schools, does not even cover the cost of students’ tuition [15]. Additionally, DACA recipients are not eligible for Federal Student Loans and although some Federal Scholarship and Loan Forgiveness programs do exist, they are open only to US citizens or US nationals, making DACA recipients ineligible [16]. Additionally, US public schools generally offer different tuition rates for in-state versus out-of-state tuition. However half of the states in the US allow for public schools to charge DACA students out-of-state tuition, even when they would be eligible for in-state tuition if they were documented [17]. Despite these barriers, DACA students identified several sources of support that facilitated their matriculation into medical school. This support came both in the form of advice from other DACA medical students, advice from administrators and advocates within both undergraduate and medical schools, and emotional and financial support from mentors and family.

DACA medical students were acutely aware of the differences in their experiences in medical school compared to the average American medical student. Several of them described the cognitive dissonance of attending their “white coat” ceremonies during the same weeks as the Trump administration’s announcement that DACA would be rescinded. Others described the difficulty of maintaining focus and motivation for their studies when they were unsure that they would be able to graduate or apply to residency. Medical school has already been identified as an acutely stressful period and these students had extra concern regarding their futures and the future and legal status of themselves and their family members. Despite this additional stress, the students remained resilient and adaptable, describing how they habituated to the additional stress and uncertainty they faced and remained focused on their goals of becoming physicians.

Medical school is a known stressor with symptoms of emotional disturbance found in approximately 30% of medical students both in the US and abroad [18–21]. Due to their unique position coupled with their tenuous immigration status, DACA medical students may experiences even greater stress. All of the DACA medical students described some level of anxiety regarding the future and identified the uncertainty surrounding the DACA program as a source of this anxiety that added to the typical emotional turmoil of medical school. They all wanted to become practicing physicians, but felt obligated to have some level of alternative plans outlined in case DACA was rescinded and they were unable to practice in the United States. Several stated that they would attempt to become physicians abroad, while some indicated that they would look into a career in consulting work if they were unable to practice medicine. Ultimately however, all of the medical students we spoke to indicated that they would prefer to practice medicine in the United States so that they could contribute to the American medical system and support people within their communities of origin and American patients alike.

### Limitations

This analysis has several limitations including the relatively small, convenience sample of DACA students who completed the survey and interview. However, given estimates of 70–100 DACA students in medical school currently, these numbers represent a respectable portion of the community. It is possible that some DACA medical students were apprehensive to complete an in-depth interview due to fear of their status being compromised or publicized, which could explain the low interview numbers. While MCAT and GPA scores were reported by participants, most students did not take the STEP1/STEP2 exams yet and these scores were not available. This information would have provided additional measures to compare the academic performance of the DACA sample. Further, only five survey participants agreed to participate in the interview portion of the analysis thereby limiting the ability to reach full data saturation. As such, this work is considered preliminary and will inform future studies in this area.

## Conclusions

The current study highlights the unique experiences of medical students in the United States who are also DACA recipients. In the face of considerable barriers and uncertainty throughout their lives and academic careers, participants describe how such personal hardships have motivated them to pursue a rigorous and competitive career in order to better serve their communities and society at large. DACA recipient medical students do not represent the good immigrant story but rather what comprehensive immigration policy can make possible by allowing individuals to pursue self-actualization free from the threat of detention and deportation.

## Supplementary Information

**Additional file 1.**

**Additional file 2.**

## Data Availability

The datasets used and analyzed during the current study are available from the corresponding author on reasonable request.

## Abbreviations

DACA: Deferred Action for Childhood Arrivals
US: United States
GPA: Grade point averages
MCAT: Medical College Admissions Test
AAMC: Association of American Medical Colleges
SD: Standard deviation
PCP: Primary Care Physician
