# Supplementary material for: Deferred Action for Childhood Arrivals (DACA) medical students – an examination of their journey and experiences as medical students in limbo
Source: BMC Med Educ. 2021 Jun 28;21:358. doi: 10.1186/s12909-021-02787-5 (PMC8240215; doi:10.1186/s12909-021-02787-5)
Supplement: Supplementary file 1 — Additional file 1. [file 12909_2021_2787_MOESM1_ESM.pdf]

# DACA Med Instrument

BACKGROUND:

President Donald Trump has given an ultimatum to Congress in hopes to find a more permanent legislative solution to Barack Obama's executive order, Deferred Action for Childhood Arrivals (DACA), by March 5, 2018. This announcement was made by Attorney General Jeff Sessions on September 5, 2017. In his remarks, Sessions states DACA-eligible individuals whose permit becomes expired after the aforementioned date will lose all protections from deportation and lose their authorization for employment.

There is an estimated undocumented population in the United States of approximately 11 million people. Of those, about 800,000 are enrolled in the DACA program. DACA served as a temporary solution for a specific subset of individuals who underwent intense vetting that revealed no significant record of criminal activity, and had to prove a minimum level of education. Given these premises, we can only speculate about the immense positive effects the undocumented community could have if they received similar protections.

GOALS OF THIS STUDY:

The positive effects of DACA on undocumented young people have been hypothesized in various studies, but never quantified in terms of impact on the US society. The end of DACA would significantly and negatively impact the career path of these individuals, forcing them out of the physician workforce, as well as the US healthcare system.

The purpose of this study is to elucidate the value that current undocumented/DACA medical students provide to healthcare and its growing, diverse patient population. You are being asked to take part in a research study because you are an undocumented/DACA medical student currently studying in the United States.

Being in a research study is completely voluntary. You can choose not to be in this research study. You can also choose to participate now and change your mind later.

If you agree to take part in this research, you will be asked to complete an anonymous questionnaire regarding your educational background and current status in medical school, among other information. Your participation in this study will take about 10 minutes, or the time required to complete the survey. We will not be able to trace back to individual respondents; consequently, there will be no further actions from you once your questionnaire is submitted. We expect that 60 people will take part in this research study. Only aggregate statistics will be presented to the scientific community.

You can choose not to answer any question you do not wish to answer. You can also choose to stop taking the survey at any time. You must be at least 18 years old to participate. If you are younger than 18 years old, please stop now.

The possible risks to you in taking part in this research are:

Possible discomfort due to current uncertainty of DACA

Potential loss of confidentiality of data

The possible benefits to you for taking part in this research are:

There are no potential benefits to participants

To protect your identity as a research subject, no identifiable information will be collected, the research data will not be stored with identifiers, and the researcher(s) will not share your information with anyone. In any publication about this research, your name or other private information will not be used.

If you have any questions about this research, please contact the Dr. Emanuela Taioli at emanula.taioli@mountsinai.org. You can also call the Program for the Protection of Human Subjects Office at 212-824-8200.

## Background Information

| | | |
|---|---|---|
| 1 | Which gender do you identify as? | ◯ Female<br>◯ Male<br>◯ Trans-Female<br>◯ Trans-Male |
| 2 | What is your age in years? | ________________ |
| 3 | What is your ethnicity? | ◯ Latino/Hispanic<br>◯ Non-Latino/Hispanic |
| 4 | What is your race? [Check all that apply] | ☐ Black/ African American<br>☐ White<br>☐ Asian/Pacific Islander<br>☐ Native American/ American Indian<br>☐ Other |
| | Other: | ________________ |

5 What is your country of birth?

- ◯ Afghanistan
- ◯ Albania
- ◯ Algeria
- ◯ Andorra
- ◯ Angola
- ◯ Antigua and Barbuda
- ◯ Argentina
- ◯ Armenia
- ◯ Aruba
- ◯ Australia
- ◯ Austria
- ◯ Azerbaijan
- ◯ The
- ◯ Bahrain
- ◯ Bangladesh
- ◯ Barbados
- ◯ Belarus
- ◯ Belgium
- ◯ Belize
- ◯ Benin
- ◯ Bhutan
- ◯ Bolivia
- ◯ Bosnia and Herzegovina
- ◯ Botswana
- ◯ Brazil
- ◯ Brunei
- ◯ Bulgaria
- ◯ Burkina Faso
- ◯ Burma
- ◯ Burundi
- ◯ Cambodia
- ◯ Cameroon
- ◯ Canada
- ◯ Cabo Verde
- ◯ Central African Republic
- ◯ Chad
- ◯ Chile
- ◯ China
- ◯ Colombia
- ◯ Comoros
- ◯ Republic of the
- ◯ Costa Rica
- ◯ Cote d'Ivoire
- ◯ Croatia
- ◯ Cuba
- ◯ Curacao
- ◯ Cyprus
- ◯ Czechia
- ◯ Denmark
- ◯ Djibouti
- ◯ Dominica
- ◯ Dominican Republic
- ◯ Ecuador
- ◯ Egypt
- ◯ El Salvador
- ◯ Equatorial Guinea
- ◯ Eritrea
- ◯ Estonia
- ◯ Ethiopia
- ◯ Fiji
- ◯ Finland
- ◯ France
- ◯ Gabon
- ◯ The
- ◯ Georgia
- ◯ Germany
- ◯ Ghana
- ◯ Greece
- ◯ Grenada

◯ Guatemala
◯ Guinea
◯ Guinea-Bissau
◯ Guyana
◯ Haiti
◯ Holy See
◯ Honduras
◯ Hong Kong
◯ Hungary
◯ Iceland
◯ India
◯ Indonesia
◯ Iran
◯ Iraq
◯ Ireland
◯ Israel
◯ Italy
◯ Jamaica
◯ Japan
◯ Jordan
◯ Kazakhstan
◯ Kenya
◯ Kiribati
◯ South
◯ Kosovo
◯ Kuwait
◯ Kyrgyzstan
◯ Laos
◯ Latvia
◯ Lebanon
◯ Lesotho
◯ Liberia
◯ Libya
◯ Liechtenstein
◯ Lithuania
◯ Luxembourg
◯ Macau
◯ Macedonia
◯ Madagascar
◯ Malawi
◯ Malaysia
◯ Maldives
◯ Mali
◯ Malta
◯ Marshall Islands
◯ Mauritania
◯ Mauritius
◯ Mexico
◯ Micronesia
◯ Moldova
◯ Monaco
◯ Mongolia
◯ Montenegro
◯ Morocco
◯ Mozambique
◯ Namibia
◯ Nauru
◯ Nepal
◯ Netherlands
◯ New Zealand
◯ Nicaragua
◯ Niger
◯ Nigeria
◯ North Korea
◯ Norway
◯ Oman
◯ Pakistan
◯ Palau
◯ Palestinian Territories
◯ Panama
◯ Papua New Guinea

- ◯ Paraguay
- ◯ Peru
- ◯ Philippines
- ◯ Poland
- ◯ Portugal
- ◯ Qatar
- ◯ Romania
- ◯ Russia
- ◯ Rwanda
- ◯ Saint Kitts and Nevis
- ◯ Saint Lucia
- ◯ Saint Vincent and the Grenadines
- ◯ Samoa
- ◯ San Marino
- ◯ Sao Tome and Principe
- ◯ Saudi Arabia
- ◯ Senegal
- ◯ Serbia
- ◯ Seychelles
- ◯ Sierra Leone
- ◯ Singapore
- ◯ Sint Maarten
- ◯ Slovakia
- ◯ Slovenia
- ◯ Solomon Islands
- ◯ Somalia
- ◯ South Africa
- ◯ South Korea
- ◯ South Sudan
- ◯ Spain
- ◯ Sri Lanka
- ◯ Sudan
- ◯ Suriname
- ◯ Swaziland
- ◯ Sweden
- ◯ Switzerland
- ◯ Syria
- ◯ Taiwan
- ◯ Tajikistan
- ◯ Tanzania
- ◯ Thailand
- ◯ Timor-Leste
- ◯ Togo
- ◯ Tonga
- ◯ Trinidad and Tobago
- ◯ Tunisia
- ◯ Turkey
- ◯ Turkmenistan
- ◯ Tuvalu
- ◯ Uganda
- ◯ Ukraine
- ◯ United Arab Emirates
- ◯ United Kingdom
- ◯ Uruguay
- ◯ Uzbekistan
- ◯ Vanuatu
- ◯ Venezuela
- ◯ Vietnam
- ◯ Yemen
- ◯ Zambia
- ◯ Zimbabwe

| | | |
|---|---|---|
| 6 | What state in the US do you consider your homestate? | ◯ Alabama<br>◯ Alaska<br>◯ Arizona<br>◯ Arkansas<br>◯ California<br>◯ Colorado<br>◯ Connecticut<br>◯ Delaware<br>◯ Florida<br>◯ Georgia<br>◯ Hawaii<br>◯ Idaho<br>◯ Illinois<br>◯ Indiana<br>◯ Iowa<br>◯ Kansas<br>◯ Kentucky<br>◯ Louisiana<br>◯ Maine<br>◯ Maryland<br>◯ Massachusetts<br>◯ Michigan<br>◯ Minnesota<br>◯ Mississippi<br>◯ Missouri<br>◯ Montana<br>◯ Nebraska<br>◯ Nevada<br>◯ New Hampshire<br>◯ New Jersey<br>◯ New Mexico<br>◯ New York<br>◯ North Carolina<br>◯ North Dakota<br>◯ Ohio<br>◯ Oklahoma<br>◯ Oregon<br>◯ Pennsylvania<br>◯ Rhode Island<br>◯ South Carolina<br>◯ South Dakota<br>◯ Tennessee<br>◯ Texas<br>◯ Utah<br>◯ Vermont<br>◯ Virginia<br>◯ Washington<br>◯ West Virginia<br>◯ Wisconsin<br>◯ Wyoming |
| 7 | How would you define your hometown where you spent most of your childhood? [Check all that apply] | ☐ Disadvantaged<br>☐ Ethnically Diverse<br>☐ Working Class<br>☐ Tech-town<br>(Check all that apply) |
| 7a | How would you define your hometown's socioeconomic status? | ◯ Low Income<br>◯ Middle Income<br>◯ High Income |
| 7b | How would you define your hometown's location? | ◯ Urban<br>◯ Rural |

| | | |
|---|---|---|
| 8 | What language(s) do you speak at home? [Check all that apply] | ☐ English<br>☐ Spanish<br>☐ French<br>☐ Chinese (Mandarin)<br>☐ Chinese (Cantonese)<br>☐ Tagalog<br>☐ Vietnamese<br>☐ Arabic<br>☐ Korean<br>☐ Russian<br>☐ Other<br>(Check all that apply) |
| 8a | If Other please specify: | __________________________ |
| 9 | How many people are in your household excluding yourself? | __________________________ |
| 10 | What was your annual household income before taxes last year? | ◯ Less than $20,000<br>◯ $20,000 to $39,999<br>◯ $40,000 to $59,999<br>◯ $60,000 to $79,999<br>◯ $80,000 to $99,999<br>◯ Over $120,000 |
| 11 | Do your parents have health insurance? | ◯ Yes ◯ No |
| 12 | Do you have health insurance? | ◯ Yes ◯ No |
| 12a | How do you receive your health insurance? | ◯ Employer<br>◯ Institution<br>◯ Private<br>◯ Other |
| 13 | Have you worked with disadvantaged populations of any kind? | ◯ Yes ◯ No |
| 13a | What populations have you worked with? [Check all that apply] | ☐ Homeless<br>☐ LGBT<br>☐ Elderly<br>☐ Impoverished/low income<br>☐ Ethnic Minorities<br>☐ Physically Disabled<br>☐ Developmentally Disabled<br>☐ Mentally Ill<br>☐ Other<br>(Check all that apply) |
| 13b | Please briefly describe your involvement below: | __________________________ |

## Undergraduate Education

14 What school did you graduate from for your undergraduate degree? __________________________________

15 What year did you graduate?

○ 2017 ○ 2016 ○ 2015
○ 2014 ○ 2013 ○ 2012
○ 2011 ○ 2010 ○ 2009
○ 2008 ○ 2007 ○ 2006
○ 2005 ○ 2004 ○ 2003
○ 2002 ○ 2001 ○ 2000

16 What month did you graduate?

○ January ○ February
○ March ○ April ○ May
○ June ○ July ○ August
○ September ○ October
○ November ○ December

17 What was your undergraduate major?

◯ Africana Studies ◯ Ancient History
◯ Anthropology ◯ Architecture
◯ Astronomy ◯ Biochemistry
◯ Biology ◯ Biophysics
◯ Chemistry ◯ Cinema and Media Studies
◯ Classical Studies ◯ Cognitive Science
◯ Communication ◯ Comparative Literature
and Theory ◯ Criminology
◯ Earth Science ◯ East Asian Languages
and Civilizations ◯ Economics
◯ Engineering Major ◯ English
◯ Environmental Studies
◯ Fine Arts ◯ French and Francophone
Studies ◯ Sexuality and Women's Studies
◯ German ◯ Health and Societies
◯ Hispanic Studies ◯ History
◯ History of Art ◯ International Relations
◯ Italian Studies ◯ Jewish Studies
◯ Latin American and Latino Studies
◯ Linguistics ◯ Mathematics
◯ Modern Middle Eastern Studies
◯ Music ◯ Near Eastern Languages and
Civilizations ◯ Nutrition Science
◯ Philosophy ◯ Physics
◯ Political Science ◯ Psychology
◯ Religious Studies ◯ Romance Languages
◯ Russian and East European Studies
◯ Sociology ◯ South Asia Studies
◯ Theater Arts ◯ Urban Studies
◯ Visual Studies ◯ Other

Other: ______________________________

If you double majored, what was your second major?

◯ Africana Studies ◯ Ancient History
◯ Anthropology ◯ Architecture
◯ Astronomy ◯ Biochemistry
◯ Biology ◯ Biophysics
◯ Chemistry ◯ Cinema and Media Studies
◯ Classical Studies ◯ Cognitive Science
◯ Communication ◯ Comparative Literature and Theory ◯ Criminology
◯ Earth Science ◯ East Asian Languages and Civilizations ◯ Economics
◯ Engineering Major ◯ English
◯ Environmental Studies
◯ Fine Arts ◯ French and Francophone Studies ◯ Sexuality and Women's Studies
◯ German ◯ Health and Societies
◯ Hispanic Studies ◯ History
◯ History of Art ◯ International Relations
◯ Italian Studies ◯ Jewish Studies
◯ Latin American and Latino Studies
◯ Linguistics ◯ Mathematics
◯ Modern Middle Eastern Studies
◯ Music ◯ Near Eastern Languages and Civilizations ◯ Nutrition Science
◯ Philosophy ◯ Physics
◯ Political Science ◯ Psychology
◯ Religious Studies ◯ Romance Languages
◯ Russian and East European Studies
◯ Sociology ◯ South Asia Studies
◯ Theater Arts ◯ Urban Studies
◯ Visual Studies ◯ Other

18 What was your undergraduate minor? [if applicable]

- ◯ Africana Studies
- ◯ Ancient History
- ◯ Anthropology
- ◯ Architecture
- ◯ Astronomy
- ◯ Biochemistry
- ◯ Biology
- ◯ Biophysics
- ◯ Chemistry
- ◯ Cinema and Media Studies
- ◯ Classical Studies
- ◯ Cognitive Science
- ◯ Communication
- ◯ Comparative Literature and Theory
- ◯ Criminology
- ◯ Earth Science
- ◯ East Asian Languages and Civilizations
- ◯ Economics
- ◯ Engineering Major
- ◯ English
- ◯ Environmental Studies
- ◯ Fine Arts
- ◯ French and Francophone Studies
- ◯ Sexuality and Women's Studies
- ◯ German
- ◯ Health and Societies
- ◯ Hispanic Studies
- ◯ History
- ◯ History of Art
- ◯ International Relations
- ◯ Italian Studies
- ◯ Jewish Studies
- ◯ Latin American and Latino Studies
- ◯ Linguistics
- ◯ Mathematics
- ◯ Modern Middle Eastern Studies
- ◯ Music
- ◯ Near Eastern Languages and Civilizations
- ◯ Nutrition Science
- ◯ Philosophy
- ◯ Physics
- ◯ Political Science
- ◯ Psychology
- ◯ Religious Studies
- ◯ Romance Languages
- ◯ Russian and East European Studies
- ◯ Sociology
- ◯ South Asia Studies
- ◯ Theater Arts
- ◯ Urban Studies
- ◯ Visual Studies
- ◯ Other

Other: ______________________________

19 What was your undergraduate GPA?

______________________________
(Please write out to two decimal places)

20 How did you finance your undergraduate education? [Check all that apply]

- ☐ Personal loans
- ☐ State aid
- ☐ Family contribution
- ☐ Scholarships
- ☐ Grants
- ☐ Personal financing (i.e. personal job income)
- ☐ Other

(Check all that apply)

20a Please briefly elaborate if other:

__________________________________

21 Do you have outstanding loans from your undergraduate education?

◯ Yes ◯ No

21a How much is your outstanding loan?

__________________________________

(Please round to the nearest hundredth)

22 During your undergraduate education, were you involved in: [Check all that apply]

- ☐ Volunteer work
- ☐ Research
- ☐ Mentorship programs

(Check all that apply)

22a Please describe the work you did in each of the categories you selected above:

__________________________________

(Please limit answers to 2-3 sentences)

## Graduate Education

23 Did you get a graduate degree prior to starting medical school?

◯ Yes ◯ No

23a What school did you attend for your graduate degree?

__________________________________

23b What year did you graduate?

- ◯ 2017
- ◯ 2016
- ◯ 2015
- ◯ 2014
- ◯ 2013
- ◯ 2012
- ◯ 2011
- ◯ 2010
- ◯ 2009
- ◯ 2008
- ◯ 2007
- ◯ 2006
- ◯ 2005
- ◯ 2004
- ◯ 2003
- ◯ 2002
- ◯ 2001
- ◯ 2000

23c What month did you graduate?

- ◯ January
- ◯ February
- ◯ March
- ◯ April
- ◯ May
- ◯ June
- ◯ July
- ◯ August
- ◯ September
- ◯ October
- ◯ November
- ◯ December

23d What was your field of study/ what degree did you get?

__________________________________

23e Do you have outstanding loans from your graduate education?

◯ Yes ◯ No

23f How much is left in your outstanding loan?

__________________________________
(Please round to the nearest hundredth)

## Medical School

24 Where do you attend medical school?

__________________________________

25 What program are you currently enrolled in? [Check all that apply]

- ☐ MD
- ☐ PhD
- ☐ MPH
- ☐ MBA
- ☐ DO
- ☐ Other

25a Other:

__________________________________

26 What year are you in?

- ◯ First Year
- ◯ Second Year
- ◯ Third Year
- ◯ Fourth Year
- ◯ Research Gap Year

27 What year did you take the MCAT?

- ◯ 2017
- ◯ 2016
- ◯ 2015
- ◯ 2014
- ◯ 2013
- ◯ 2012
- ◯ 2011
- ◯ 2010
- ◯ 2009
- ◯ 2008
- ◯ 2007
- ◯ 2006
- ◯ 2005
- ◯ 2004
- ◯ 2003
- ◯ 2002
- ◯ 2001
- ◯ 2000

(Enter most recent year if taken multiple times)

---

28 What month did you take the MCAT?

- ◯ January
- ◯ February
- ◯ March
- ◯ April
- ◯ May
- ◯ June
- ◯ July
- ◯ August
- ◯ September
- ◯ October
- ◯ November
- ◯ December

---

29 What was your total MCAT score?

________________________________

---

30 How many times did you take the MCAT?

________________________________

---

31 During your application cycle prior to matriculation, how many medical schools did you apply to?

________________________________

---

32 How many schools invited you for an interview?

________________________________

---

33 How many schools offered you an acceptance?

________________________________

---

34 Which schools were you accepted to?

________________________________

---

35 Of the medical schools you applied to, how many were unclear or opaque about their policies on accepting DACA students?

________________________________

(Please give numerical value)

---

35a Please explain:

________________________________

| | | |
|---|---|---|
| 36 | How are you financing your medical education? [Check all that apply] | ☐ Personal loans<br>☐ State aid<br>☐ Family contribution<br>☐ Scholarships<br>☐ Grants<br>☐ Personal financing (i.e. personal job income)<br>☐ Other<br>(Check all that apply) |
| 36a | Please elaborate: | __________________________ |
| 37 | Currently, what residency training program are you planning on pursuing? | ◯ Anesthesiology<br>◯ Dermatology<br>◯ Emergency Medicine<br>◯ Family Medicine<br>◯ General Surgery<br>◯ Internal Medicine<br>◯ Medicine<br>◯ Medicine-Pediatrics<br>◯ Neurological Surgery<br>◯ Neurology<br>◯ Obstetrics/Gynecology<br>◯ Opthamology<br>◯ Oral Maxillofacial Surgery<br>◯ Orthopaedic Surgery<br>◯ Osteopathic Neuromusculoskeletal Medicine<br>◯ Otolaryngology<br>◯ Pathology<br>◯ Physical Medicine and Rehabilitation<br>◯ Plastic Surgery<br>◯ Preventative Medicine<br>◯ Proctology<br>◯ Psychiatry<br>◯ Radiation<br>◯ Thoracic Surgery<br>◯ Urology<br>◯ Vascular Surgery<br>◯ Other: |
| 37a | Please elaborate: | __________________________ |
| 38 | Are you planning to focus in pediatrics or adults? | ◯ Pediatrics<br>◯ Adults<br>◯ Geriatrics |
| 37b | What type of Radiation medicine? | ◯ Diagnostic Radiology<br>◯ Interventional Radiology<br>◯ Nuclear Medicine<br>◯ Radiation Oncology |
| 38 | Will you be applying to a residency program in the USA? | ◯ Yes ◯ No |
| 39 | What specialty are you planning to pursue? | __________________________ |

39b Please elaborate on the reason:

________________________________
(Please limit answer to 2-3 sentences)

41 Where are you interested in practicing?
- ◯ Rural
- ◯ Urban
- ◯ Suburban

42 What type of practice are you interested in?
- ◯ Private Practice
- ◯ Research
- ◯ Academic Medicine
- ◯ Hospital Clinician
- ◯ Private Sector
- ◯ Other sector (consulting, public health, etc)
- ◯ Other

43 Do you want to practice medicine in the US? ◯ Yes ◯ No

44 Please briefly describe why you want to become a physician:

________________________________
(Please limit response to 3-4 sentences)

44a If you would like to, please feel free to attach your personal statement.

**Migration**

**These questions are optional; you can still submit your completed questionnaire above without this section if you wish.**

46 How many years have you lived in the US?

________________________________

47 If you are unable to complete a residency in the US, will you consider practicing medicine in another country? ◯ Yes ◯ No

48 Which region would you like to practice in?
- ☐ Europe
- ☐ Central/South America
- ☐ Africa
- ☐ Asia
- ☐ Australia

49 If you would like to be contacted for a potential future qualitative study, please include your email address so that we can reach you.

________________________________
