# Supplementary material for: Deferred Action for Childhood Arrivals (DACA) medical students – an examination of their journey and experiences as medical students in limbo
Source: BMC Med Educ. 2021 Jun 28;21:358. doi: 10.1186/s12909-021-02787-5 (PMC8240215; doi:10.1186/s12909-021-02787-5)
Supplement: Supplementary file 2 — Additional file 2. [file 12909_2021_2787_MOESM2_ESM.pdf]

DACA In-depth Interview Guide

**Pre-Discussion Orientation**<br><br>*NOTE: Facilitators introduce themselves and describe what an in-depth interview is and how it works. Tell respondents that the interview will last approximately 60 minutes. Acknowledge the discussion will be audiotape-recorded and provide assurances that the information that is shared with us will be used for research purposes, but only their words will be shared, not their personal information. In other words it will be like taking an anonymous survey. Ensure that each participant understands and has provided consent to the audio recording of the discussion.*
**INTRODUCTION** (NOTE: Text written using lay language)<br><br>**Interviewer:** Thank you again for completing the initial DACA study and for providing your email to answer some additional questions for this qualitative study. The information that you and others provide will be an important step towards informing the public and policy makers about your experiences.<br><br>***Do you have any questions about the study or the focus group before we get started?***<br><br>***May we turn on the tape recorder now?***

Brainstorming Topics to Cover, Please add or modify as needed<br><br>***Question ideas: https://reimaginingmigration.org/moving-stories-interview-questions-my-story/***<br><br>***Powerful videos: https://www.phdreamers.org/***

Before I start with the more open-ended questions, I'd like to just make sure I have the information correct from your survey.	Follow-up questions:<br>• Confirm that the person completed the initial DACA survey.<br>• Medical school, Year of graduation
**ICE BREAKER:** What would you be doing right now if you weren't talking to me? What classes are you taking this semester?	
**Future Career Plans**	
Please tell us about your future career plans, including medical profession and if there are any specific populations you want to work with	Follow-up questions:<br><br>• Residency / Specialty plans<br>• Any difficulty in choosing next steps because of DACA status?

	● Plan on working with underserved populations?<br>● If so, why?<br>● Are you apprehensive about the future as an undocumented physician?<br>● Do you intend to practice in a specific geographic area (urban / rural)?<br>● Please describe your goals in medicine<br>● How has DACA impacted your trajectory in medical school?
**Medical School**	
Please tell me about your decision to go to medical school	<u>Follow-up questions:</u><br><br>● How did you decide on what medical schools to apply to?<br>● How did you decide on which medical school to enroll in?<br>● What were your motivations to go to medical school?<br>● Did DACA status influence the decision?<br>● Did you have an alternative path if you did not get into medical school?
Please tell us about your experience in medical school	<u>Follow-up questions:</u><br><br>● What concerns did you have entering medical school?<br>● Do you feel it has been different than other students?<br>● Are your classmates and professors aware of your immigration status?<br>● Do you feel you don't have access to the same resources that other medical students do?<br>● Do you feel you have a support group / social network / integrate with other classmates?<br>● Do you feel that here are people in your support group who can empathize with what you're going through?<br>● Has your academic performance been affected by uncertainty in the DACA program?<br>● Are you at risk of losing your loans if DACA is terminated?<br>Will you continue medical school if DACA is terminated?

**Migration**	
Please tell us about and your family's experience migrating to the United States	<u>Follow-up questions:</u><br><br>● What was your parent's motivation for migrating? Did they choose the United States for a specific reason? What were your family's hopes?<br>● What did it feel like when you first arrive in the US?<br>● Were you with other family members or friends?
How did receiving DACA status make an impact on your life?	<u>Follow-up questions:</u><br>● What do you think your life would look like now if it had not been for DACA?<br>● How would loosing DACA status change your life?<br>● Experience of any interactions with the US government<br>● Does anyone else in your family have DACA status?<br>● Given the turbulence with DACA, would you participate in future programs offered by the government?<br>● Looking back, would you disclose your DACA status again if given the choice?
**Living in the United States**	
Please tell us about your experience living in the United States	<u>Follow-up questions:</u><br>● Were you or your parents able to find work?<br>● Did you ever have concerns for your safety or that of your family due to your DACA status?<br>● Were you aware of any bigotry/violence against you?<br>● If so, how did that impact your view of the US and Americans?<br>● How does that experience inform how you plan to practice medicine?<br>● Did you feel part of a particular community in the United States?
We've reached the end of the discussion. Is there anything else we haven't covered that you want to mention or talk about? **Is there anything you wish you could say specifically to those who do not support**	**Thank you for your time and your valuable input!**

**DACA or more permanent protections for childhood arrivals?**	
